# Assessing an Improved Protocol for Plasma microRNA Extraction

**DOI:** 10.1371/journal.pone.0082753

**Published:** 2013-12-23

**Authors:** Inés Moret, Dolors Sánchez-Izquierdo, Marisa Iborra, Luis Tortosa, Ana Navarro-Puche, Pilar Nos, José Cervera, Belén Beltrán

**Affiliations:** 1 Instituto de Investigación Sanitaria del Hospital La Fe, Valencia, Spain; 2 CIBEREHD, CIBER de enfermedades hepáticas y digestivas, Barcelona, Spain; 3 Genomics Unit, Hospital Universitari i Politècnic La Fe, Valencia, Spain; 4 Gastroenterology Unit, Hospital Universitari i Politècnic La Fe, Valencia, Spain; 5 Genetics Department, Hospital Universitari i Politècnic La Fe, Valencia, Spain; Baylor College of Medicine, United States of America

## Abstract

The first step in biomarkers discovery is to identify the best protocols for their purification and analysis. This issue is critical when considering peripheral blood samples (plasma and serum) that are clinically interesting but meet several methodological problems, mainly complexity and low biomarker concentration. Analysis of small molecules, such as circulating microRNAs, should overcome these disadvantages. The present study describes an optimal RNA extraction method of microRNAs from human plasma samples. Different reagents and commercially available kits have been analyzed, identifying also the best pre-analytical conditions for plasma isolation. Between all of them, the column-based approaches were shown to be the most effective. In this context, miRNeasy Serum/Plasma Kit (from Qiagen) rendered more concentrated RNA, that was better suited for microarrays studies and did not require extra purification steps for sample concentration and purification than phenol based extraction methods. We also present evidences that the addition of low doses of an RNA carrier before starting the extraction process improves microRNA purification while an already published carrier dose can result in significant bias over microRNA profiles. Quality controls for best protocol selection were developed by spectrophotometry measurement of contaminants and microfluidics electrophoresis (Agilent 2100 Bioanalyzer) for RNA integrity. Selected donor and patient plasma samples and matched biopsies were tested by Affymetrix microarray technology to compare differentially expressed microRNAs. In summary, this study defines an optimized protocol for microRNA purification from human blood samples, increasing the performance of assays and shedding light over the best way to discover and use these biomarkers in clinical practice.

## Introduction

microRNAs (miRNAs) comprise a family of highly conserved small non-coding RNAs (∼ 22 nt), that regulate gene expression at the post-transcriptional level. Discovered in 1993, these endogenous non-coding transcripts, represents approximately 1–2% known genes in eukaryotes and function to negatively regulate gene expression by repression or degradation through base-pairing to target mRNAs [1], [2]. microRNAs play a critical role in many biological processes such as cell proliferation and maturation, apoptosis, regulation of chronic inflammation and development of cancer [3]. Numerous studies have focused on identifying altered expression of miRNAs associated with disease and they have been proposed as candidate biomarkers [4]. In this context, in many disease including autoimmunity diseases (IBD, rheumatoid arthritis…), where there is a complex interplay of key immune and non-immune cells elements [1], miRNAs emerge as important immune regulators and its impact on the development or prevention of disease is under study [3], [5].

In blood samples, it is well recognized that circulating miRNAs are either packaged in microparticles (exosomes, microvesicles and apoptotic bodies) or associated with RNA-binding proteins [Argonaute 2 (Ago2)] or lipoprotein complexes (high-density lipoprotein (HDL)) [6]–[10]. The extremely small size of miRNAs renders most conventional biological amplifications tools less effective. Also, the close similarities among family members of miRNAs have presented challenges for developing miRNA-specific detection assays. In addition, it has been observed that during the purification process, small RNAs could be less efficiently precipitated in alcohol solutions. Therefore, owing to the uniqueness of miRNAs distinct from the protein-coding mRNAs, there are differences in the approaches to detect and quantify miRNAs. [11], [12]. All this means that a valid method for extracting and analyzing microRNAs still remains to be found.

To address this issue, several studies have tried to develop different approaches [13], [14]. However, the results in terms of accurate quantifications and measurements are still far from being the most suitable. The correct identification of disease-related miRNA patterns from body fluids remains to be elucidated [4].

Other problems related to the very short length and low levels of microRNAs in such fluidic samples make problematic to obtain efficient and reproducible microRNA recovery. In the qPCR approach, it is common to test only a few numbers of molecules. The short structure of such PCR targets may affect the efficiency of the amplification process, including adding bias to the quantification of miRNA expression. In addition, it is reported that RNA purified from plasma can contain inhibitors that may also affect qPCR efficiency.

Since the first reports revealing the presence of miRNAs in plasma and serum [15]–[18], many researchers have focused on identifying the microRNA profile in these effortless samples. If these blood microRNAs could serve as biomarkers, patients could be better monitored and easily managed by clinicians. With the advent of more complete screening protocols where precious clinical samples are being analyzed for a variety of biomarkers, the importance of reduced sample requirements is increasing, generating a need for extremely sensitive and reproducible assays. In addition, a valuable biomarker requires robust and reproducible assays working in clinically available samples as well as in archived material. Therefore, in an attempt to identify the most suitable method for extracting microRNA from human plasma samples, we have tested the RNA isolation efficiency of three different available RNA isolation protocols and extraction reagents: mirVana (Ambion, Life Technologies), TRIzol-LS (Invitrogen) and miRNeasy Serum/Plasma Kit (Qiagen). We have tested different protocols to identify which one offers more effectiveness in clinical samples preparation. The suitability of adding a carrier has also been tested. FlashTag miRNA direct labeling from Genisphere, together with miRNA 3.0 microarray from Affymetrix, have been used to test the efficiency of selected extraction protocols by detecting the highest number of miRNA molecules.

## Materials and Methods

### Study Population

Twenty consecutive peripheral blood samples from newly diagnosed of inflammatory bowel disease patients at the Gastroenterology Department of the Hospital Universitari i Politècnic La Fe (Valencia, Spain) and healthy volunteer controls were collected for the study. The control group comprised patients who were undergoing an ileocolonoscopy but who did not met a clinicopathological suspicion of inflammation, such as polyps, and resulted in normal colonoscopy. Blood samples were collected prior to any therapeutic procedure. Biopsies from bowel mucosa were obtained at the time of the ileocolonoscopy. All the subjects gave their written informed consent on the day of recruitment. The study was approved by the Ethical Committee on Clinical Research of the Hospital Universitari i Politècnic La Fe (ref: 2010/0468) in compliance with the Declaration of Helsinki.

### Sample Material and Preparation

Anticoagulated blood (K3-EDTA) was obtained after 12 hours of fasting. Two different approximations were followed to obtain plasma samples: 1) Blood was carefully layered onto Histopaque 1077 (Sigma-Aldrich, UK) at RT and centrifuged at 213 g 30 min (without brakes). The upper-layer phases containing the white cell-rich plasma were removed and cells and platelets were subsequently separated from the plasma by spinning down at 2375 g 10 min. At this point, plasma aliquots were either processed immediately for total RNA isolation or stored at −80°C for later extractions. 2) Other protocols [2] with some modifications were also used, including fresh blood kept on ice until being centrifuged directly in a 15 mL corning-like tube of at 1700 g for 10 min. The plasma layer was then centrifuged at 2000 g for 10 min and supernatant was used fresh.

### RNA Extraction Methods

Three different commercial kits/reagents were employed to test which was most suitable for obtaining RNA from human plasma samples: mirVana PARIS kit (Cat n°AM1556, Ambion, Life Technologies, Texas, USA), TRIzol-LS (Cat n°10296010, Ambion, Life Technologies, Carlsbad, CA, USA ) and miRNeasy Serum/Plasma Kit (Cat n°.217184. Qiagen, Hilden, Germany). Two different approximations were applied in mirVana kit: one following the exact manufacturer’s instructions and the other, a modified version of the centrifugation step to 30 min at 4°C after the phenol/chloroform step in order to increase the final RNA yields. In both cases, a starting volume of 400 µL of thawed plasma or freshly isolated plasma was used. Total RNA was eluted into 100 µL of pre-heated (95°C) RNase-free water.

RNA was also extracted from plasma using the TRIzol-LS reagent. From frozen plasma, 4 aliquots of 400 µL each were used and 3,5 mL from freshly isolated plasma. In all cases, RNA extraction was done according to the commercial protocol, including glycogen addition before isopropanol and an overnight precipitation step. In addition, a combined version of TRIzol-LS extraction followed by purification with mirVana column kit was employed [2], [19]. In this protocol, RNA was eluted in 100 µL of pre-heated (95°C) RNase-free water.

Finally, for the Qiagen kit assay, two 200 µL of plasma aliquots were used to get enough final RNA volume to perform all quality tests and miRNA expression determinations. RNA was eluted with 14 µL of RNase-free water according the manufacturer’s instructions and the elution’s volumes from the same sample were pooled to obtain a final volume of 28 µL.

In addition, RNA extracted from biopsies using the mirVana kit was employed as a control of the whole RNA recovery process and microarray detection. All biopsy samples selected for further microarray tests were obtained from the same plasma patients or donors, thereby being paired.

### Carrier Addition in RNA Purification Process

Due to the difficulty in quantifying RNA in plasma samples, and with the aim of RNA yield increase, purified Torulla yeast RNA carrier (Ambion®, Life Technologies) was included in trial processes at two different concentrations: 10 µg/mL (as previously reported) [2] and 1 µg/mL (as the low amount). Carrier was added before the chloroform protocol step. A spike in control was also included in some samples that underwent the Qiagen protocol if it necessary to check extraction by qPCR later on.

### RNA Quality and Integrity

RNA purity and concentration were evaluated by spectrophotometry using NanoDrop ND-2000 (ThermoFisher). Initially, only the A260/230 and A260/280 ratios were used to assess the presence of contaminants: peptides, phenols, aromatic compounds, or carbohydrates and proteins. In the end, however, the full spectrum was considered because absorbance was also detected in other wavelengths.

Quality and the related size of total and small RNA was assessed by the Agilent 2100 Bioanalyzer microfluidics-based platform (Agilent Technologies, Santa Clara, USA) with two chips: Agilent RNA 6000 Nano Kit for total RNA and Agilent Small RNA kit for low molecular weight RNA. Electropherograms were visualized using the Agilent 2100 Expert software which included data collection, peak detection and interpretation of the different profiles.

### miRNA Expression Levels Measured by Microarrays

All plasma RNA preparations assessed by microarray technology were those obtained from duplicate starting material (200 µL and 200 µL of plasma) using the miRNeasy kit to control for variation in the RNA preparation steps, loading the aqueous phase onto a single affinity column and mixing the final elution volume (14 µL each) in a single tube.

A total of 14 samples from patients and donors were hybridized following the manufacturer protocols: 4 plasma samples (PL), 2 plasma samples with carrier at standard previously reported 10 µg/ml concentration (PLsc) and 4 plasma samples with carrier at ten-fold lower 1 µg/ml concentration (PLlc) and 4 biopsies (BIO). For biopsies, 500 ng of total RNA were added to the labelling mix. Despite the previously determined concentration, 8 µL of plasma and plasma with carrier RNA were labeled. FlashTag miRNA direct labelling (Genisphere) was used following the manufacturer’s procedures. The GeneChip® Scanner 3000 7G System and reagents from Affymetrix were used to hybridize, wash, stain and scan the arrays.

The latest version of the miRNA Affymetrix platform miRNA 3.0 based on mirBase version 17 [20] was used to obtain the miRNA profile. This version contains 19,913 probesets including 5818 human premature, cajal body associated (sca), small nuclear organizers (sno) and mature miRNA.

### Statistical Analysis

Expression console free software from Affymetrix was used for quality control and assessment of probe set normalized values. Partek Genomic Suite 6.6 version (Partek Incorporated, St. Louis) was used for microarray statistical analysis, PCA and HC generation. Following the miRNA expression workflow, normalization of data included RMA background correction, quartile normalization, log2 transformation values and median polish according to Genisphere indications for the FlashTag Biotin labelling kit. ANOVA test at the different categories was performed and FDR and p-values were applied to generate the list of more significant probe values. GraphPad software (San Diego, CA) was employed for t-test statistical analysis.

All microRNA data described in this manuscript have been deposited in NCBI’s Gene Expression Omnibus Database (http://www.ncbi.nlm.nih.gov/geo/). The accession number at GEO is GSE51375.

## Results

**Figure 1:**
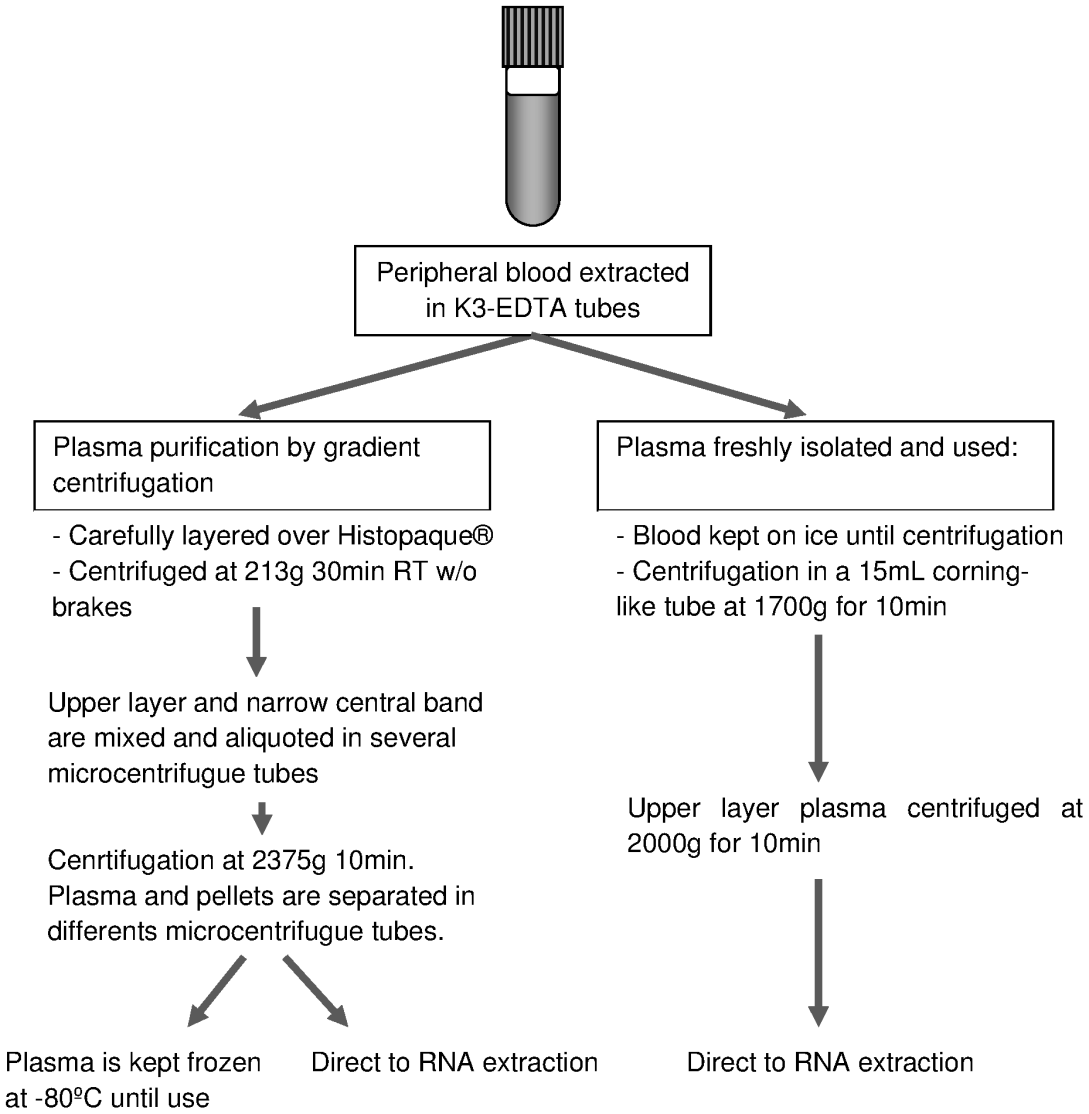
Flow chart of the different plasma processing methods.

The different pre-analytical protocols employed for plasma purification are briefly explained in figure 1. The addition of an extra centrifugation step to pellet cells (2375 g 10 min) also resulted in significant platelet reduction (elements that might interfere in later analysis) of plasma samples (data not shown). Another interfering factor, such as hemolysis, that could affect the differential miRNA profiles identification, was also determined in our plasma samples. The measurements of hemolysis by Harboe method [21] resulted in values (0.021±0.002 g/L) inside the established normal range (from 0.01 to 0.03 g/L) indicating that this potential confounding factor should not interfere in later miRNA detection. This effect was also controlled by checking common described hemolytic related miRNA levels [4], [22] in microarray results (data not shown).

**Figure 2:**
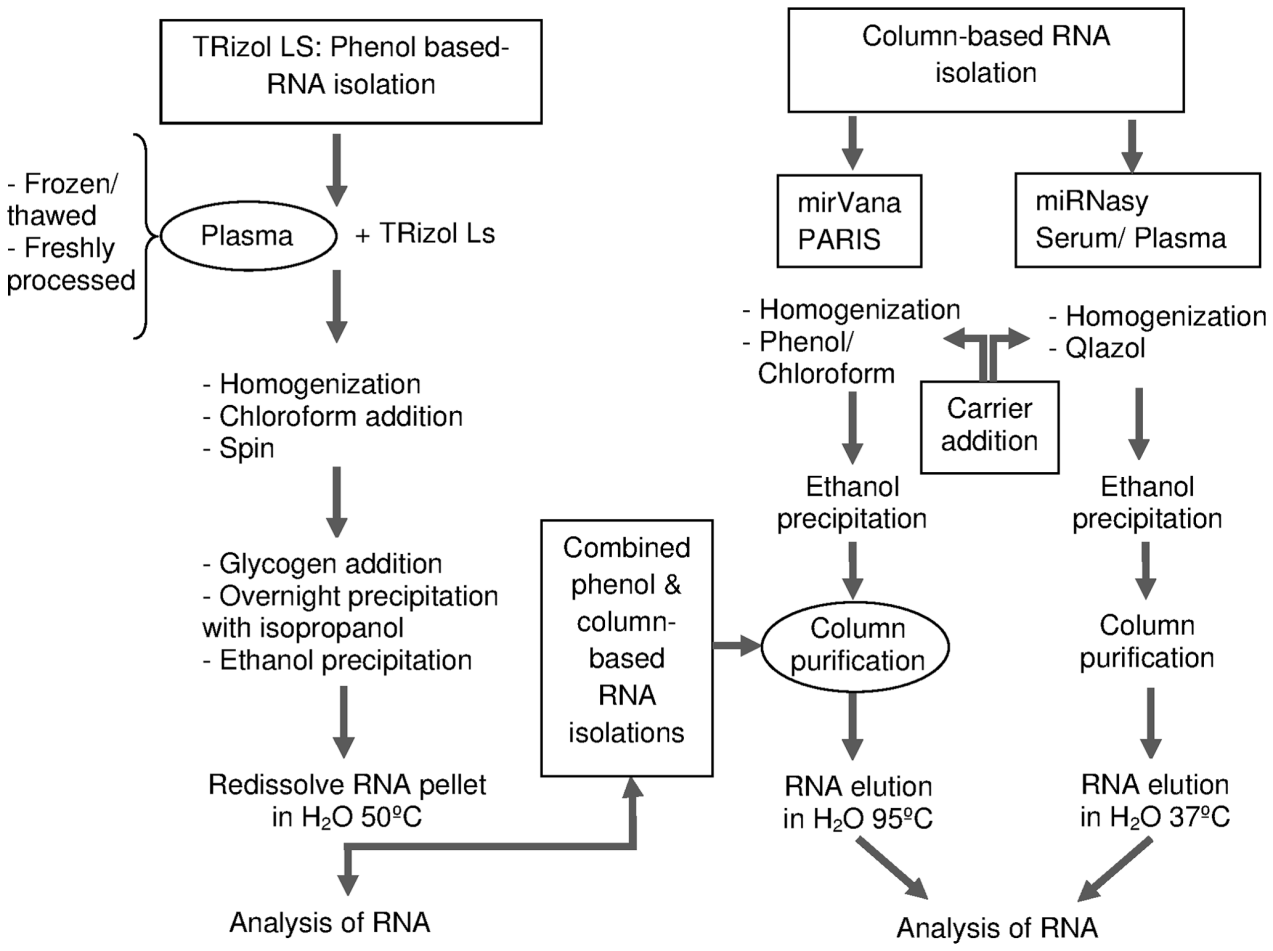
Flow chart of the different total RNA extraction methods.

**Figure 3:**
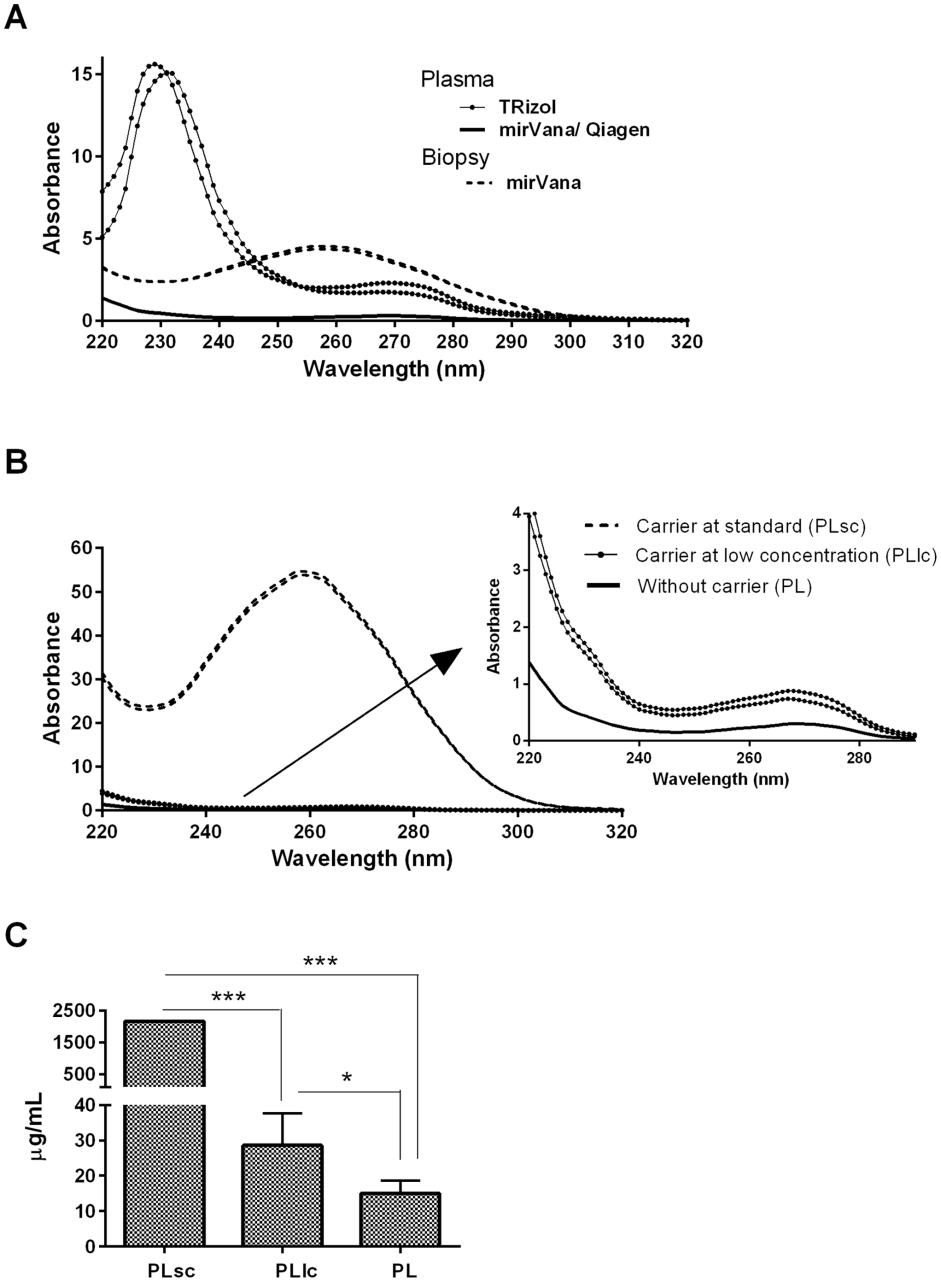
NanoDrop results from the different methods employed. A) RNA 220–320 absorbance spectra from the different samples analyzed and protocols employed. B) NanoDrop spectra from samples under miRNA easy extraction. The effect of carrier addition at different doses is also depicted. C) RNA quantification by NanoDrop. Samples without carrier (PL), carrier at low concentration (1 µg/mL, PLlc) and carrier at standard concentration (10 µg/mL, PLsc), all RNA extracted with the Qiagen kit, was analyzed by NanoDrop technology. ***stands for *p*<0.001 and *for *p*<0.05.

After that, RNA was extracted using different isolation methods (Figure 2) including three different commercially available kits and reagents. In all cases, RNA was eluted in RNase free H_2_O and kept frozen until analysis. The presence of contaminants was assessed by NanoDrop spectrophotometry. In terms of quantity and purity, no better results were obtained from fresh plasma than from frozen plasma (Table 1). As shown in figure 3.A, low peaks at 260/280 nm region were observed for the commercial kits: mirVana and miRNeasy. Between them, miRNeasy produced a higher concentration (3-fold) in the eluted RNA (Table 1). Higher 260 nm peaks, related to higher RNA quantity, were observed in TRizol-LS extracted RNA, together with highest organic and phenolic contaminants (Figure 3.A), thus excluding those samples from further studies. In an effort to increase the purity of Trizol based extracted samples, a combination of this phenolic reagent followed by matrix based column purification (mirVana) was used but still resulted in lower RNA quality data than mirVana alone (Table 1). Because plasma aliquots were either processed immediately for total RNA isolation or frozen for later extractions, it was possible to test the effect of prolonged storage time on the RNA extraction yield. Frozen samples constantly gave rise to high concentrated total RNA and RNA yield, independently of the protocol/kit employed for total RNA isolation (Table 1). The increased RNA yield could be due to the different centrifugation conditions (Figure 1) for fresh and frozen samples, conditions established in our lab for the first, and previously published [2] for the second: blood kept on ice and spun down with high speed but low centrifugation time.

**Table 1:** NanoDrop spectrophotometer results from the different RNA extraction protocols: total amount, concentration and 260/280 ratios.

Method		RNA amount (µg/mL)	RNA yield (µg/1 mL plasma)	OD 260/280
Trizol LS:	fresh plasma	49.9±24.95	2.85	1.57±0.11
	frozen plasma	60.14±43.0	7.52	1.68±0.13
mirVana:	fresh plasma	2,45±0.21	0.61	1.99±0.10
	frozen plasma	5.39±3.14***	1.33	1.61±0.40
TRizol/mirVana:	fresh plasma	1.87±0.71	0.05	1.77±0.29
	frozen plasma	3,88±3,11***	0.24	1,08±0.79
miRNeasy:	frozen plasma	15,00±3,70	1,05	1,48±0.17

*p*<0.001 between each marked sample and miRNeasy. Mean plus SD.

**Figure 4:**
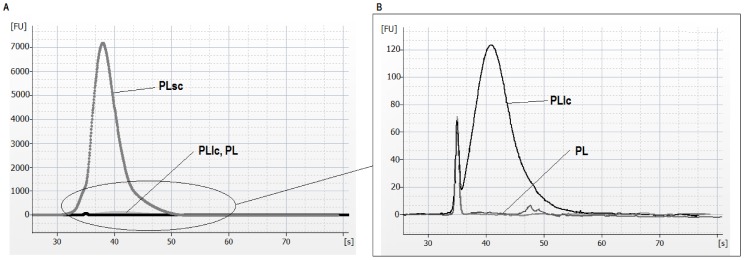
Bioanalyzer analysis of total RNA from plasma extractions. RNA isolated with different methods was analyzed using RNA Small Kit in an Agilent 2100 Bioanalyzer. The electropherograms of miRNA easy extractions show the size distribution in nucleotides (nt) and fluorescence intensity (FU) of total RNA in plasma at two scales: A) Plasma with standard carrier concentration (10 µg/mL PLsc), B) Plasma with low carrier concentration (1 µg/mL PLlc) and without carrier (PL).

By using 260/280 nm absorbance ratio RNA purity was assessed. The different extraction methods gave rise mainly to poor 260/280 ratios (Tables 1 and 2). In order to improve recovery and quantification of microRNAs from plasma, the benefit of adding an RNA carrier (yeast RNA) before RNA extraction was also evaluated. As depicted in figure 3.B and 3.C, and table 2, carrier addition increased both quantity and purity of extracted RNA (PLsc and PLlc vs PL). However, the carrier amounts were determinant in RNA yield, revealing that higher doses of carrier (10 µg/mL for PLsc) can mask the real amount of extracted nucleic acid because the presence of the carrier itself in the elution volume, and making more difficult to accurately quantify (Figure 3.B and 3.C, Table 2) and to analyze the integrity of the RNA (figure 4). In spite of this, lower doses of carrier (1 µg/mL for PLlc) helped to improve the quantity of Nanodrop application (Figure 3.B and 3.C) and the Bioanalyzer test, thus helping to assess the validation process (Figure 4).

**Table 2:** Comparative table on the carrier effect on the total RNA yielded, measured by NanoDrop spectrophotometer, and its purity.

Method	RNA amount (µg/mL)	RNA yield (µg/1 mL plasma)	OD 260/280
mirVana w/o carrier	5.39±3.14	1.33	1.61±0.40
mirVana+carrier (10 µg/mL)	355.3±5.20	88.83	2.13±0.50
miRNeasy w/o carrier	15.00±3.70	1.05	1.48±0.17
miRNeasy+carrier (10 µg/mL)	2161.9±22.20	151.33	2.04±0.01

Mean plus SD.

**Figure 5:**
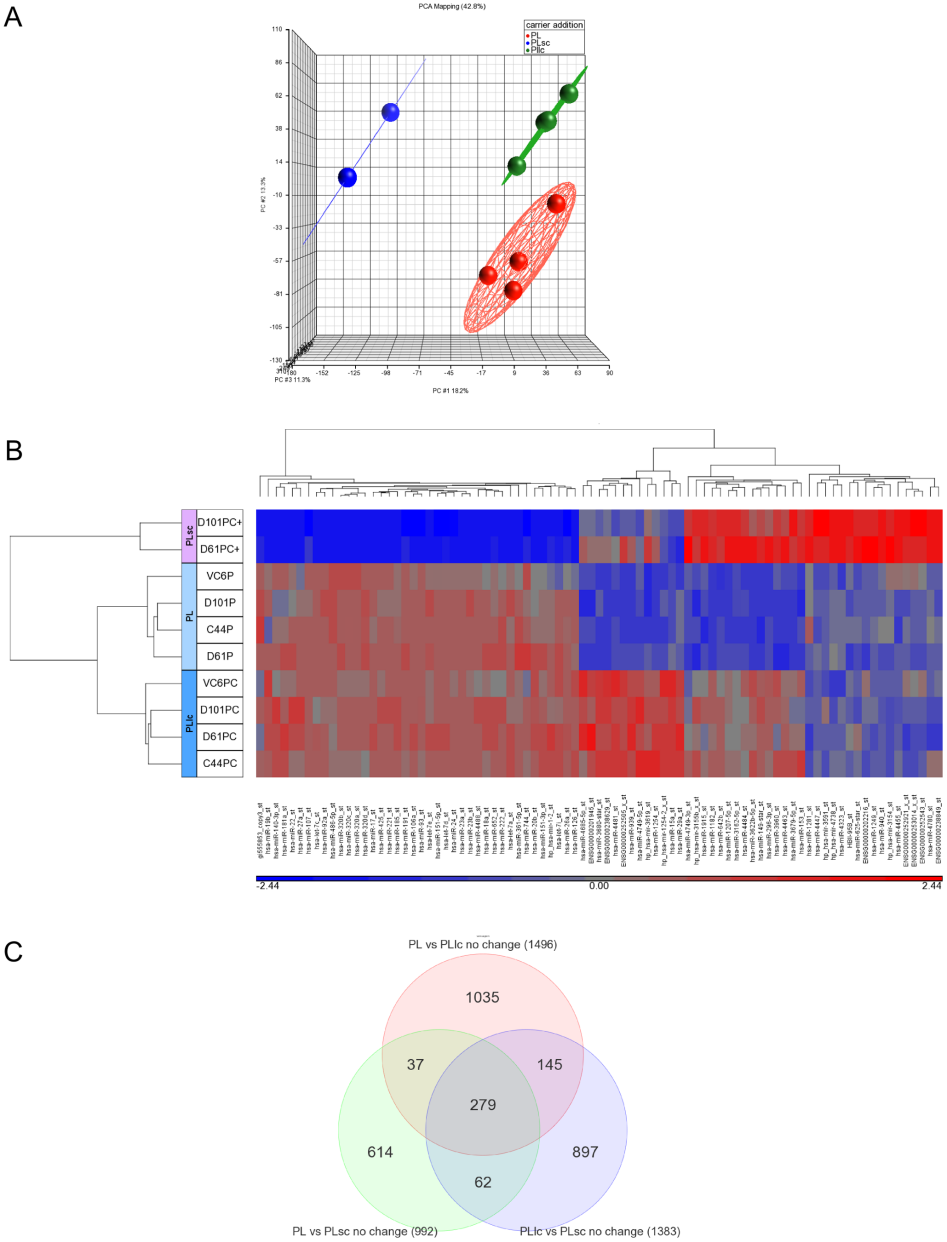
miRNA expression profiles defined by microarrays technology. A) PCA scatter plot of normalized data from plasma samples. B) Hierarchical clustering showing microRNA expression profiles by means of ANOVA test FDR p0.05, differentially expressed in the three categories: without carrier (PL) and with carrier at low (1 µg/mL PLlc) or standard concentration (10 µg/mL PLsc). C) Venn diagram comparing the number of overlapping miRNAs targets among the set of plasma samples. List was generated by ANOVA contrast of each category and a fold-change of 2 to −2 and a p-value ≤0.05.

Nowadays, microarray technology is allowing detection of a higher number of miRNA molecules. Thus, 14 biopsies and samples with no carrier or the two different carrier concentrations indicated above were tested. Comparing broadly expressed miRNA levels obtained by miRNA Affymetrix microarray technology, all values for BIO, PL and PLlc were very similar but not PLsc (Figure S1.A and S1.B, Table S1). As quality control (QC) assessment is crucial before any analysis is performed, we checked that recommended parameters for homogeneous results were into the expected values (Table S2). Furthermore, analysis of miRNA reported to be released by erythrocytes showed similar expression between samples [4] through carrier addition at the exception of PLsc, demonstrating that this factor is crucial is miRNA levels at the tested sample (data not shown). Due to the high cost of microarray technology and the low performance of a higher amount of carrier addition constantly seen in quality assays, only two PLsc amongst all samples analyzed by NanoDrop and Bioanalyzer, were assayed using this technology. PLsc rendered clearly different miRNA profile than the same patient PLlc or PL plasma sample (Figure S2, Table S3). On the contrary, BIO or PLlc showed closely related expressed miRNA profile (Figure S3). PCA indicated that miRNA expression profiles were similar in PLlc or PL samples, so all were closer in the 3D PCA plot (Figure 5.A). According to this, diversity in signal profiles is more related to the individual characteristics of patient and control samples rather than to the addition of a small amount of carrier, but, despite of this, detected levels of each molecule is severely affected by the introduction of carrier at previously published concentrations [2]. Furthermore, in all processed samples, detection of low expressed miRNAs was improved, facilitating quality control tests. On the contrary, whole array intensity was clearly knockdown in no carrier PL samples (Table S1). Different general intensity for each array regarding the type of sample assayed is reduced by normalization array algorisms, but even after RMA and background correction procedures recommended by Affymetrix, the main differences can still be detected.

Differences between PLsc RNA and all other conditions samples were confirmed in clustering maps (Figure 5.B) that show the relative expression of all detected miRNAs: high doses of carrier were detrimental when comparing the different microRNAs profiles. In order to analyze expression patterns under different carrier conditions from microarray profiling results, we generated an overlapping Venn diagram highlighting those similar expressed molecules between the different set of samples (Table S4). As shown in figure 5.C, the Venn diagram displays that 1496 microRNAs are commonly detected between PL and PLlc. However, PLsc results in different microRNAs profile than PL. Only 992 miRNA are detected in both set of samples.

Total RNA from 4 biopsies from same patients and donors (BIO) for plasma study were extracted with mirVana protocol, employing them as an example of good quality RNA for microarray experiments and to compare expressed miRNAs that could be related afterwards to its presence in the plasma from those individuals. BIO samples were very useful as expression controls of some characteristic miRNA molecules being homogeneously expressed in all but the PLsc sample (Figure S2, S3).

## Discussion

Human plasma has been a rich source of biomarker discovery [23]. The study of microRNAs in biological fluids such as plasma and serum has rapidly expanded, signaling their possible application in clinics, thereby avoiding the use of invasive diagnostics techniques in patients. If pathological entities are characterized by aberrant miRNA expression in plasma, identification of miRNA signatures for specific diseases could achieve an early and differential diagnosis [24]. However, the utility of those stable molecules as key biomarker requires a highly reliable and reproducible protocol for RNA purification. The accurate and robust measurement of miRNA in biological fluids has been challenging due to a number of factors including the very short and variable nucleotide sequence and the low amount of molecules present in those samples [25]. Taking into account all these aspects, in this work we have first tested pre-purification steps for RNA isolation that, in our opinion, could later affect microRNA detection (Figure 1, 2). The steps required for sample preparation seem to be critical. Blood has to be processed immediately after venipuncture and plasma should undergo two consecutive centrifugation steps to eliminate PBMC and platelets contaminants respectively. Measurements of hemolysis in plasma samples should be done, prior to RNA extraction, to detect and be aware of possible interferences with specific miRNAs associated with hemolysis [26]. Standardizing the sample procedure for plasma purification may not be easy to follow in daily clinical practice but effectiveness in avoiding these contaminants is important to obtain stable results. The aim of minimizing cellular content in plasma samples is to avoid unwanted bias in miRNA profiles originated by cellular contaminants [2]. Considering also that in plasma samples, a disease-specific signature might be overwhelmed by microRNAs contained in platelets [25], the inclusion of an extra centrifugation step seems to be mandatory. Furthermore, the pelleted PBMC can be frozen and employed in further analysis. Our observations, apart from confirming all those addressed issues, also demonstrate that miRNAs are well preserved in frozen plasma samples so RNA can be extracted later on.

In this study we have tested different commercial kits or combinations of them to define the most suitable microRNA isolation protocol for human plasma samples (Figure 2). To identify prerequisites for accurate and representative measurement of miRNAs profiles in biological fluids such as plasma [27], we have focused on RNA extraction and purification steps. Specific kits for plasma and serum extraction have been developed recently. The performance of the miRNeasy plasma/serum kit from Qiagen relays in low volume elution matrix column, thus obtaining a more concentrated sample (Figure 3). Quality ratios may vary, but in our hands, Qiagen RNA yields are more stable through different extraction days. The lowest elution volume gives a very important advantage over other commercially available kits or reagents avoiding sample manipulation to concentrate it. The matrix column-based extraction method is highly effective, reducing contaminants in the RNA sample. Alternative extraction methods for RNA based on organic solvents such TRizol has also been tested, obtaining lower quality and performance results. Regarding to this, a recent paper enforce the idea that this method is unsuitable for miRNA isolation because small RNAs require other RNAs as carriers to increase the yield in the purification process [28] and indicating that organic solvent based RNA extraction methods must be discarded for miRNA studies.

Besides the detailed validation of the pre-analytical steps affecting miRNA purification, addition of different carrier amounts has also been tested. Our observations support the hypothesis that a lower concentration of carrier than the previously one published for total RNA extraction in plasma and serum can be more helpful in miRNA purification, increasing purity and quantity (Figure 3). It is important to point out that the quantity of carrier added to the samples was crucial in the RNA yield, indicating that commonly used RNA carrier concentration can mask the quantity of extracted RNA and affect accuracy in quantification and quality analysis (Figure 3, 4). Because no good quality assessment could be done, only two samples extracted with standard amount of carrier were tested by array in order to check their protocol performance and expression profiles (Figure S1). Both expression profiles were very similar, but clearly different from all the other sample profiles, so further studies with this type of samples were not performed (Figure 5, Tables S1–S4). Biopsies paired to the same plasma donors and patients have been employed as controls for microarray performance and expression profile comparison (Figure S2, S3). However, quality controls and intensity values for those good performing samples indicate that obtained RNA is consistently different from plasma RNA, and subsequent analysis, specially normalization and background subtraction, should be done separately.

Plasma RNA isolation procedure is important for improving yield but is also determining level of inhibitors. As we have seen, some current methods or combinations of them increase the final concentration of RNA, leading to higher levels of contaminants, therefore reducing applicability or efficiency of miRNA detection protocols. Such contaminants may be derived from biofluids or introduced by reagent carry-over during sample preparation [25]. Considering all, avoiding extra sample manipulation (such as speedvac after RNA extraction) is essential. On the other hand, some authors estimated that the addition of carrier means that the RNA in the samples cannot be accurately quantified through [18], but in plasma samples RNA is undetectable by using common molecular laboratory methodologies. So, a control of the full extraction process is mandatory due to the high cost of validation or detection techniques. We have obtained good results correlations by using lower doses of carrier (Figure 3, 4). It is commonly accepted that the improvement in microRNA detection is likely due to improved microRNA recovery in the presence of carrier [12], [18]. We have also observed, in agreement with former reported observations [26], that NanoDrop spectrophotometer quantification is unsuitable for plasma extracted RNA supporting that. Lower amounts of carrier can be the best option to optimize the extraction process. It is also strongly recommended checking the full 220–340 nm spectra for accurate definition of carryover contaminants.

Some papers reinforce the idea that PCR is the best way for microRNA detection and quantification [29]–[31]. Different amplification methods have been described using LNA or other annealing loop strategies. In our opinion, the introduction of an external miRNA control is not ridding the problem because the efficiency of amplification for such an external control sequence can differ from targets. PCR amplification can be laborious, including some preamplification steps, and the rapid increase in the number of miRNAs in the databases renders qPCR inefficient on a genomic scale, being better as a validation method instead [32], [33]. Unlike the qPCR strategies, the Affymetrix miRNA technology relies on the FlashTag biotin 3D direct labeling system and does not apply any amplification step, so each microRNA molecule is not undergoing processes that could affect the final product [32]. From our understanding, the FlashTag labeling kit is a reliable method where, as far as one microRNA molecule can be detected by microarray sensitivity, the measured expression value will be closer to the biological situation (Figure 5). On the other hand, miRNA molecules that are present at very low levels in the original plasma or serum samples are not expected to show significant or very high differences at detection, thus, their utility as biomarkers should be limited or none. In our experience, a real limitation for miRNA expression quantification relies in the extraction method that should always be performed in the same way (Figure 1, 2).

In conclusion, we have demonstrated that microRNA from RNA plasma samples obtained using the miRNeasy serum/Plasma kit and adding 10 fold lower doses of carrier than previously published gives rise to reproducible and efficient RNA extractions for later analysis by microarrays. By detecting broadly and highly expressed miRNA molecules we confirmed the efficiency and performance of those extraction conditions. Highly expressed molecules were detected at very similar levels in biopsies, plasma and plasma including a low amount of carrier samples. Plasma extracted using commonly reported carrier concentration reported very different in microarray assays so miRNA target selection must be still well validated according to different protocols for RNA extraction and detection techniques.

## Supporting Information

Figure S1 **Quality samples control of microarrays results.** A) Log expression signals after of robust multi-array average RMA, detected above background or DABG normalization. B) Relative log expression signals.
(TIF)

Figure S2 **PCA scatter plot of data for all four RNA methodology extraction categories: Biopsies (BIO), plasma with standard carrier concentration (PLsc), plasma with low carrier concentration (PLlc) and plasma without carrier (PL).**
(TIFF)

Figure S3 **Hierarchical clustering showing microRNA expression profiles of differentially expressed in the four categories.**
(TIFF)

Table S1 **Quality control results: List of intensity values generated by Expression console software, showing normalized array probes values for all plasma samples under RMA and DABG algorithms.**
(XLS)

Table S2 **Summary of quality control (QC) Affymetrix values for all array tested samples, including: Biopsies (BIO), plasma with standard carrier concentration (PLsc), plasma with low carrier concentration (PLlc) and plasma without carrier (PL).**
(XLSX)

Table S3 **List of more significant miRNA (adjusted FDR p-value <0.05) differentially expressed in all four categories.**
(XLS)

Table S4 **ANOVA contrast of each category two by two.** Lists are generated by p<0.05 and Fold Changes ≥2. Those lists were used for Venn diagram generation and overlapping lists.
(XLS)
