# Supplementary figures and images for: Assessing an Improved Protocol for Plasma microRNA Extraction
Source: PLoS One. 2013 Dec 23;8(12):e82753. doi: 10.1371/journal.pone.0082753 (PMC3871541; doi:10.1371/journal.pone.0082753)

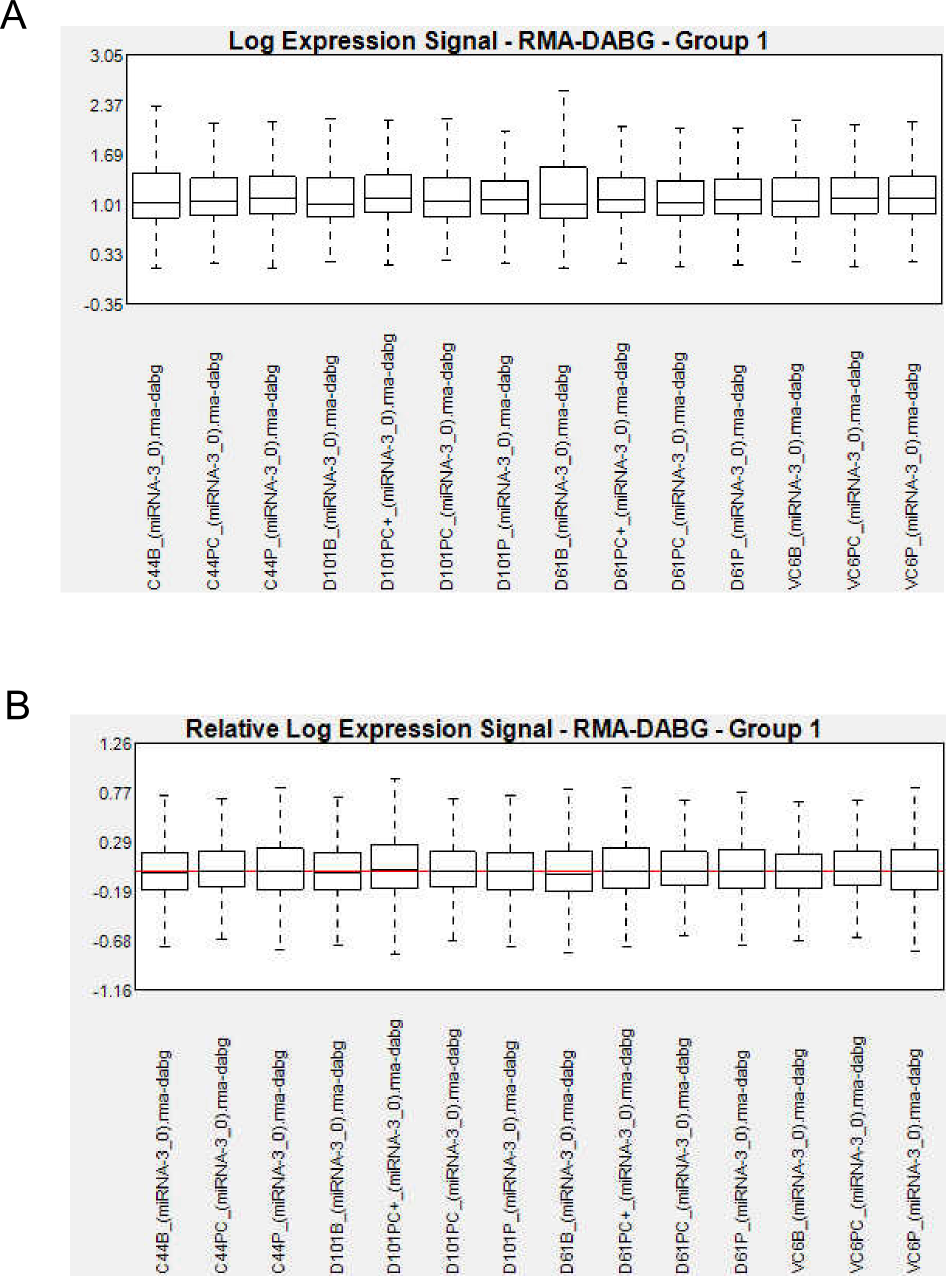

Supplement: Figure S1 — Quality samples control of microarrays results. A) Log expression signals after of robust multi-array average RMA, detected above background or DABG normalization. B) Relative log expression signals. (TIF) [file pone.0082753.s001.tif]

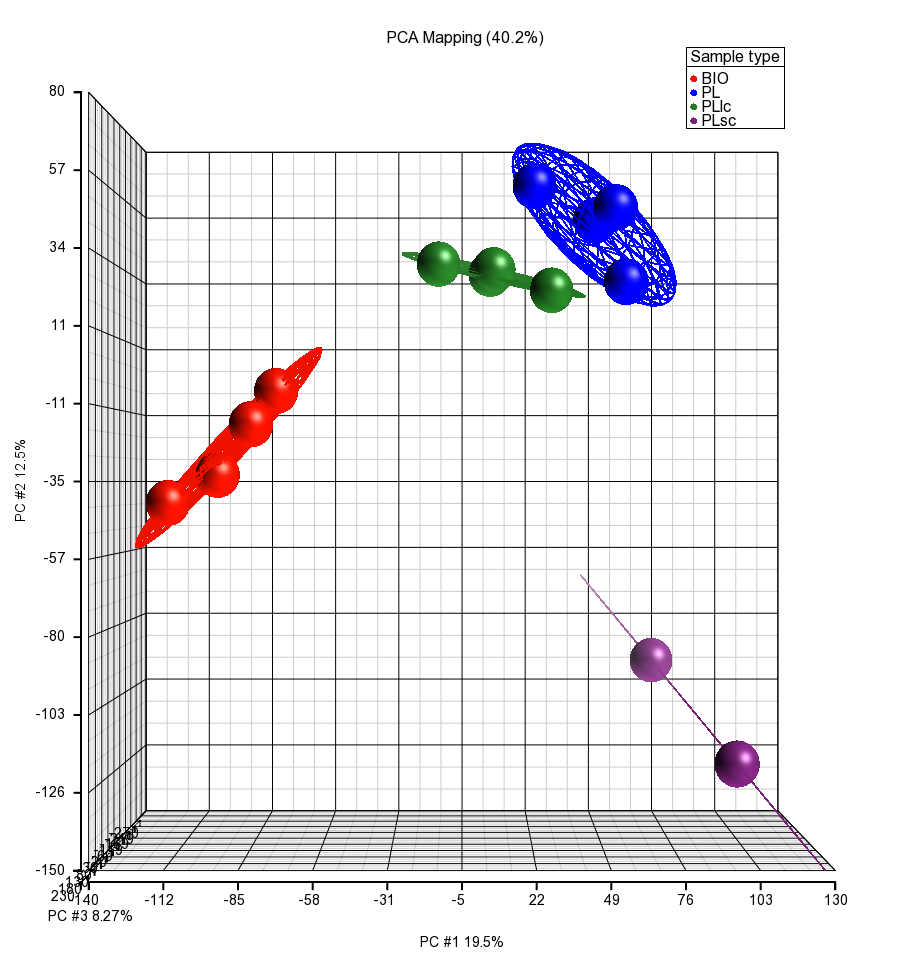

Supplement: Figure S2 — PCA scatter plot of data for all four RNA methodology extraction categories: Biopsies (BIO), plasma with standard carrier concentration (PLsc), plasma with low carrier concentration (PLlc) and plasma without carrier (PL). (TIFF) [file pone.0082753.s002.tiff]

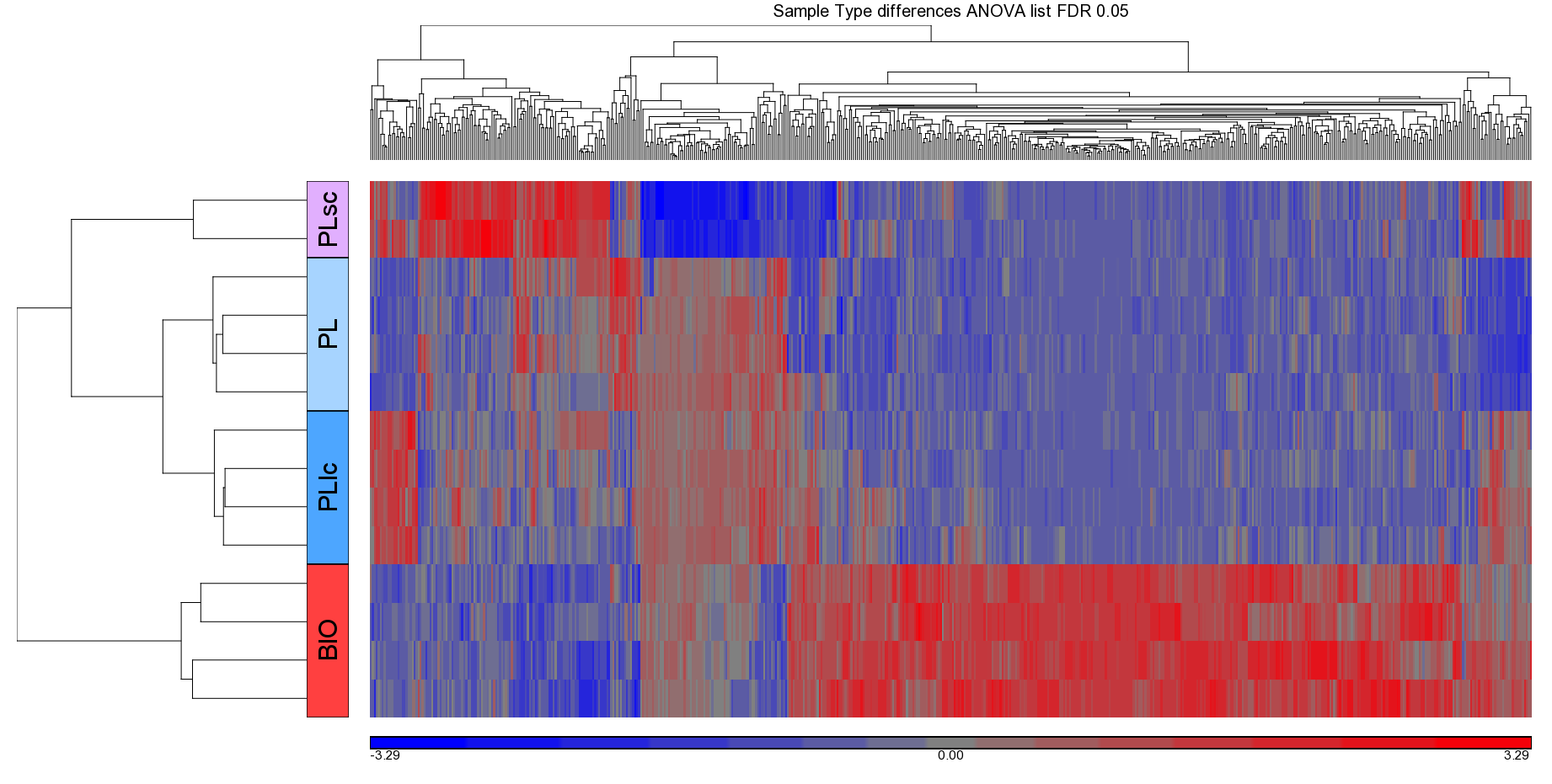

Supplement: Figure S3 — Hierarchical clustering showing microRNA expression profiles of differentially expressed in the four categories. (TIFF) [file pone.0082753.s003.tiff]
